# Bach1: Function, Regulation, and Involvement in Disease

**DOI:** 10.1155/2018/1347969

**Published:** 2018-10-02

**Authors:** Xinyue Zhang, Jieyu Guo, Xiangxiang Wei, Cong Niu, Mengping Jia, Qinhan Li, Dan Meng

**Affiliations:** Department of Physiology and Pathophysiology, School of Basic Medical Sciences, Fudan University, Shanghai 200032, China

## Abstract

The transcription factor BTB and CNC homology 1 (Bach1) is widely expressed in most mammalian tissues and functions primarily as a transcriptional suppressor by heterodimerizing with small Maf proteins and binding to Maf recognition elements in the promoters of targeted genes. It has a key regulatory role in the production of reactive oxygen species, cell cycle, heme homeostasis, hematopoiesis, and immunity and has been shown to suppress ischemic angiogenesis and promote breast cancer metastasis. This review summarizes how Bach1 controls these and other cellular and physiological and pathological processes. Bach1 expression and function differ between different cell types. Thus, therapies designed to manipulate Bach1 expression will need to be tightly controlled and tailored for each specific disease state or cell type.

## 1. Introduction: Bach1 Structure and Cellular Distribution

**Figure 1:**
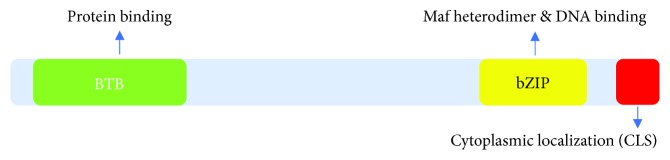
Schematic diagram of the structure of Bach1.

BTB and CNC homology 1 (Bach1) is a member of the Cap ‘n' Collar and basic region leucine zipper family (CNC-bZip) of transcription factors. It is widely expressed in mammalian tissues, and the human variant consists of 736 amino acids. The N-terminal region of Bach1 contains a BTB/POZ domain, which functions as a protein interaction motif, while the C-terminal bZip domain binds to DNA [1] and mediates the heterodimerization of Bach1 with small Maf proteins (e.g., MafF, MafG, and MafK) (Figure 1). Once formed, the Bach1-Maf heterodimers inhibit the transcription of many oxidative stress-response genes, including heme oxygenase-1 (HO-1) [2] and NADPH quinone oxidoreductase 1(NQO1) [3], by binding to Maf recognition elements (MAREs) in the gene promoters. Another transcription factor in the basic region leucine zipper family, Bach2, is expressed in B cells, T cells, macrophages, and neural cells [4] and is involved in oxidative stress-mediated apoptosis, macrophage-mediated innate immunity, and adaptive immune response [5–7].

Bach1 contains six cysteine-proline (CP) motifs, four of which are located in a heme-binding region near the C-terminus. Heme inactivates Bach1 by interacting with two of the CP motifs, leading to the exclusion of Bach1 from the nucleus [8], and by promoting HOIL-1-mediated ubiquitination and degradation [9]. Bach1 nuclear export is also triggered by the antioxidant-induced phosphorylation of a tyrosine residue (Bach1 tyrosine 486) [10] and by cadmium, which activates a cytoplasmic localization signal (CLS) located in the Bach1 C-terminus via a mechanism that requires extracellular signal-related kinase (ERK) [11]; both heme- and cadmium-induced Bach1 nuclear export signals are dependent on chromosome region maintenance 1 (Crm1) [12]. After export into the cytoplasm, Bach1 forms fiber-like structures on microtubules by colocalizing with intracellular hyaluronic acid-binding protein (IHABP), which regulates the subcellular localization of Bach1 [13]. Furthermore, human cells also express an alternative splice variant of Bach1, Bach1t, which lacks the leucine zipper domain and is constitutively nuclear, suggesting that Bach1/Maf heterodimer formation may also have an important role in Bach1 subcellular localization and activity [14].

**Figure 2:**
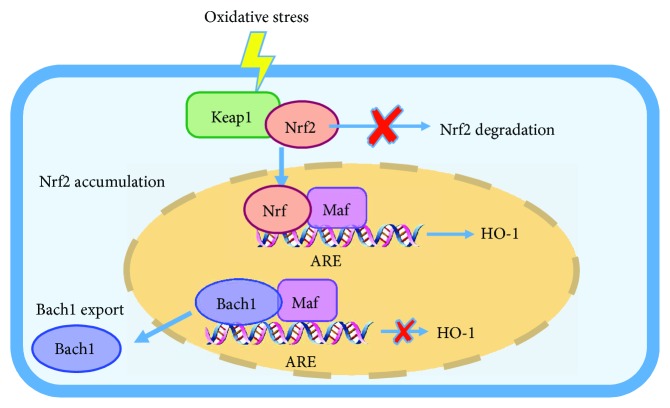
Model for competition between Nrf2 and Bach1 on MARE of HO-1 in response to oxidative stimuli. During oxidative stress, Nrf2 dissociates from Keap1 and Nrf2 degradation is inhibited, so Nrf2 will accumulate in the cytoplasm and translocate into the nucleus. Then, Nrf2 binds to MAREs as a heterodimer with small Mafs, activating HO-1 expression, while Bach1 is displaced from MAREs and exported out of the nucleus.

Bach1 competes with nuclear factor (erythroid-derived 2)-like-2 (Nrf2) for binding to the MAREs in oxidative stress-response genes. Under normal physiological conditions, nuclear Nrf2 contributes to vascular protection by inducing expression of the glutamate cysteine ligase modulatory subunit (GCLM) and the light chain component of system x_c_^−^ (xCT) in human endothelial cells, while cytoplasmic Nrf2 is bound and inhibited by Kelch-like ECH-associated protein 1 (Keap1) [15]. During oxidative stress, Nrf2 dissociates from Keap1, translocates into the nucleus, and binds to MAREs as a heterodimer with small Mafs, thereby activating oxidative stress-response genes (e.g., HO-1 and NQO1) [16], while Bach1 is displaced from MAREs and exported out of the nucleus [17] (Figure 2); evidence in hepatocytes suggests that both the nuclear import of Nrf2 and the dissociation of Bach1-MARE are promoted by sirtuin (Sirt) 6 [18]. Furthermore, a functional MARE site has been identified near the transcription start site of Bach1 transcript variant 2, and Nrf2 overexpression, as well as Nrf2-activating agents, upregulates Bach1 expression [19]. Thus, Bach1 appears to act as a functional inhibitor of Nrf2 under physiological oxygen levels [16], while Nrf2 restores Bach1 levels after oxidative stress-induced Bach1 nuclear export and degradation.

## 2. Bach1 in Oxidative Stress and Heme Homeostasis

Bach1-deficient mice are more resistant to the oxidative stresses associated with trinitrobenzene sulfonic acid- (TNBS-) induced colitis [20], hyperoxic lung injury, nonalcoholic steatohepatitis [21], and cardiovascular disease [22], as well as bleomycin-induced pulmonary fibrosis [23], while declines in Bach1 expression or activity reduced measures of oxidative stress-induced apoptosis in pancreatic *β*-cells [24] and the damaging effects of ultraviolet radiation in keratinocytes [25]. Furthermore, we have shown that Bach1 overexpression enhances the production of reactive oxygen species (ROS) from the mitochondria of endothelial cells and in the ischemic limbs of mice, which leads to increases in apoptosis and declines in angiogenesis [26]. Many of the genes targeted by Bach1 (e.g., NQO1, glutamate-cysteine ligase catalytic subunit (GCLC), glutamate-cysteine ligase modifier (GCLM), and solute carrier family 7 member 11 (SLC7A11)) [3, 27] also participate in redox regulation, including HO-1, which is essential for cell survival under conditions of oxidative stress and for the maintenance of cellular iron homeostasis in higher eukaryotes [28]. HO-1 expression is suppressed by Bach1 when heme levels are low, but higher heme levels inhibit Bach1-DNA binding and promote Bach1 nuclear export and degradation [12], thereby inducing HO-1 expression, which subsequently degrades heme while generating antioxidant molecules such as ferrous iron, carbon monoxide (CO), and biliverdin. Thus, the Bach1/HO-1 pathway forms a feedback loop that maintains heme homeostasis during periods of oxidative stress. Bach1 also appears to increase the cytotoxicity of an anticancer drug by downregulating HO-1 expression in human primary acute myeloid leukemia (AML) cells [29].

## 3. Bach1 in the Cell Cycle, Senescence, and Mitosis

The effect of Bach1 on cell proliferation and survival can differ profoundly depending on the cell type and experimental conditions. In endothelial cells, we have shown that exogenous Bach1 expression inhibited proliferation and the expression of cyclin D1 while inducing cell-cycle arrest and caspase 3-dependent apoptosis [26]; however, proliferation was also impaired in Bach1-deficient aortic smooth muscle cells (SMCs) [30], and Bach1 deficiency reduced both proliferation and activated p53-dependent senescence in murine embryonic fibroblasts under conditions of oxidative stress [31]. Notably, although many of the genes targeted by Bach1, such as E2F1, cyclin-dependent kinase 6 (CDK6), calmodulin 1 (CALM1), transcription factor binding to IGHM enhancer 3 (TFE3), EWS RNA-binding protein 1 (EWSR1), and BCL2-like 11 (BCL2L11), participate in cell-cycle control and apoptosis [27], phosphorylated Bach1 interacts with hyaluronan-mediated motility receptor (HMMR) and CRM1 to stabilize the orientation of the mitotic spindle, and depletion of endogenous Bach1 impaired spindle formation, in dividing HeLa cells [32–34]. Thus, Bach1 appears to have two distinct roles in cell proliferation, one as a transcriptional regulator of cell-cycle proteins and another during chromosomal alignment, which are dependent upon the phosphorylation state of Bach1.

In addition, Bach1 is also associated with an age-dependent loss of adaptive homeostasis. Bach1 was increased in all tissues (heart, liver, and lung) of aging mice [35] and was higher in human bronchial epithelial cells from older adults than from young adult donors [36]. Thus, Bach1 appears to attenuate redox adaptive homeostasis in aging mice and old people. Another aging-associated disease osteoarthritis is also found to be related to Bach1 due to oxidative damage. Inhibition of Bach1 by carnosic acid can induce HO-1 expression and attenuate cartilage degradation [37].

## 4. Bach1 in Angiogenesis and Myocardial Protection

**Figure 3:**
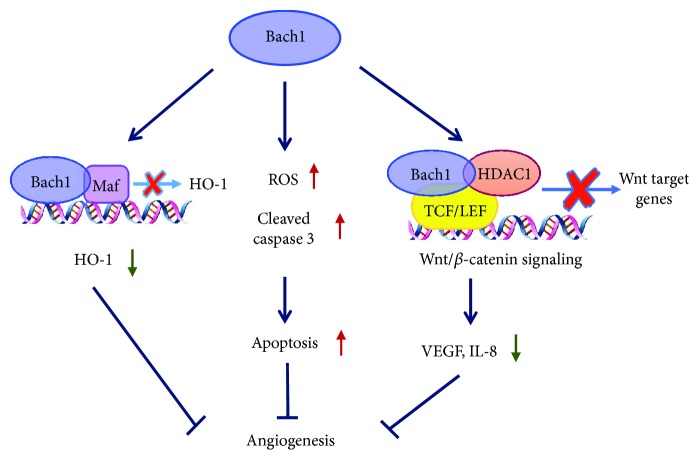
Relations between Bach1 and angiogenesis. Bach1 suppresses angiogenesis through different mechanisms. Bach1 represses the expression of HO-1, which has been shown to upregulate the expression of proangiogenic molecules (e.g., VEGF) and promote neovascularization. Bach1 overexpression also enhances the production of ROS from the mitochondria of endothelial cells, which leads to EC apoptosis and inhibits angiogenesis. Besides, Bach1 functions as a competitive inhibitor of *β*-catenin/TCF4 binding, recruits HDAC1 to the promoter of Wnt target genes, thereby reducing the expression of proangiogenic cytokines, such as VEGF and IL-8, and suppresses angiogenesis.

We have shown that measurements of perfusion, vascular density, and the expression of proangiogenic cytokines were greater in the limbs of Bach1-deficient mice than in the limbs of wild-type mice after surgically induced hindlimb ischemia [38]. Bach1 appears to limit the angiogenic response to ischemic injury, and at least one of the mechanisms by which Bach1 suppresses angiogenesis involves Wnt/*β*-catenin signaling. Canonical Wnt/*β*-catenin signaling regulates gene transcription by facilitating the transport of cytoplasmic *β*-catenin into the nucleus, where *β*-catenin forms a complex with transcription factor 4 (TCF4)/lymphoid enhancer-binding factor 1 (LEF1) and recruits transcription factors, such as CREB-binding protein (CBP), that initiate Wnt-targeted gene expression [39]. The binding of *β*-catenin also displaces histone deacetylase 1 (HDAC1) [40] and other transcriptional corepressors from TCF4 [41] and recruits transcriptional coactivators such as the histone acetyl transferase p300/CBP [42]. Bach1 functions as a competitive inhibitor of *β*-catenin/TCF4 binding, recruits HDAC1 to the promoter of TCF4-targeted genes, and prevents *β*-catenin from being acetylated by p300/CBP, thereby reducing the expression of downstream Wnt targets, such as vascular endothelial growth factor (VEGF) and interleukin- (IL-) 8, that promote angiogenic activity in human ECs [38] (Figure 3). Bach1 also appears to suppress developmental angiogenesis in zebrafish by impeding Wnt/*β*-catenin signaling and the expression of VEGF and IL-8 [43].

Arsenite stimulates angiogenesis by promoting the dissociation of Bach1 from HO-1 enhancer elements in endothelial cells [44], and the subsequent increase in HO-1 expression upregulates the expression of proangiogenic molecules such as VEGF [45]. In the heart, HO-1 expression also protects against ischemia and reperfusion injury [46], and the deletion of Bach1 upregulated HO-1, which subsequently inhibited transverse aortic constriction- (TAC-) induced left ventricular hypertrophy and remodeling [47]. However, the cardioprotective effects associated with Bach1 deficiency in mice also appear to be partially mediated by activation of the STAT3 pathway and by the inhibition of p38/MAPK signaling and apoptosis [22], and Bach1 deficiency reduces the proliferation of SMCs as well as neointimal formation in a murine model of arteriosclerosis via an HO-1-independent mechanism [30].

## 5. Bach1 in Cancer

Bach1 depletion had no effect on the growth of breast cancer cells in culture or on primary tumor growth in mice [48]; however, the expression of Bach1 and its target genes has been linked to a higher risk of breast cancer recurrence in patients [49], as well as increases in cell invasion and migration of prostate and colon cancer cells [50–52], while lower Bach1 levels have been associated with declines in breast tumor metastasis [48]. The prometastatic activity of Bach1 is at least partially mediated by increases in the expression of metastatic genes such as CXC-chemokine receptor 4 (CXCR4), high-mobility group AT-hook 2 (HMGA2), vimentin, and matrix metalloproteinases (MMPs) 1, 9, and 13 [51, 52]. Furthermore, Bach1 both suppresses and is suppressed by the metastasis-suppressor Raf kinase inhibitory protein (RKIP), and computational models suggest that this interplay between Bach1 and RKIP, as well as their downstream targets, could provide a mechanism by which environmental factors and stochastic fluctuations can trigger a metastatic phenotype in previously noninvasive cells without altering the cells' genomes [53]. Furthermore, Bach1 represses its own expression by binding to its promoter region and therefore has its own negative feedback loop [53]. This indicates that Bach1 is under tight control, and cells cannot tolerate too high expression level suggesting that Bach1 can have profound physiological effects that become deleterious when too extreme.

**Figure 4:**
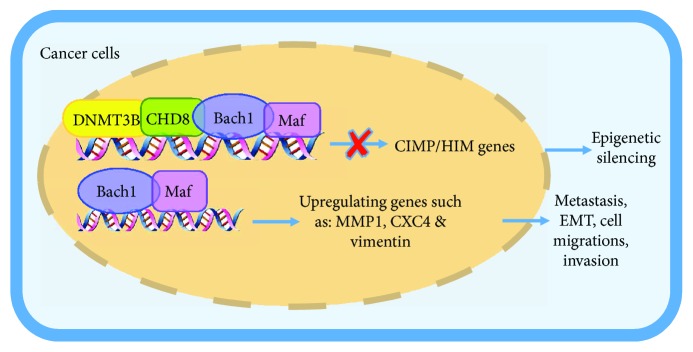
Model for genes regulated by Bach1 in cancer cells. Bach1-Maf heterodimer recruits both CHD8 and DNMT3B, leading to the hypermethylation and transcriptional silencing of tumor-suppressor genes (CIMP and HIM). Meanwhile, Bach1-Maf can increase the expression of MMP1, CXCR4, and vimentin, contributing to cancer metastasis and invasion.

Bach1 is also involved in epigenetic mechanisms of cancer progression. In both colorectal cancer and melanoma, the B-Raf protooncogene variant BRAF (V600E) upregulates v-maf avian musculoaponeurotic fibrosarcoma oncogene homolog G (MAFG), which heterodimerizes with Bach1 and recruits both chromodomain helicase DNA-binding protein 8 (CHD8, a chromatin remodeling factor) and the DNA methyltransferase DNMT3B, leading to the hypermethylation and transcriptional silencing of tumor-suppressor genes [54, 55] (Figure 4). Bach1 also promotes temozolomide (TMZ) resistance in patients with glioblastoma by antagonizing the p53-mediated suppression of O^6^-methylguanine DNA methyltransferase (MGMT); once activated, MGMT counteracts the antitumor effect of TMZ by reversing the TMZ-induced methylation of guanine residues [56].

Notably, Bach1 can also function as a tumor suppressor, and at least some of its anticancer properties can likely be attributed to its role as a regulator of HO-1 expression. In acute myeloid leukemia cells, lower levels of Bach1 expression were associated with increases in HO-1 levels and in cell viability after exposure to an anticancer drug [29], and CXCR3-B appears to inhibit the growth of breast cancer cells by promoting the nuclear localization of Bach1, which subsequently suppresses HO-1 [57]. Bach1 also suppresses the expression of transketolase (TKT), an enzyme that is required for the growth of hepatic cancer cells because it participates in the pentose phosphate pathway and in the production of the antioxidant molecule NADPH [58]. Furthermore, although oxidative stress is known to contribute to both aging and tumorigenesis and Bach1 deficiencies increase HO-1 expression, Bach1 does not appear to influence aging in mice and the rate of spontaneous tumorigenesis in p53-deficient mice and in Bach1-p53 double-deficient mice was similar [59]. Thus, Bach1 function differs between different cancer cell types, even within the same cell type (e.g., breast cancer cells), and Bach1 may have a different effect on cancer cell growth and cancer metastasis [53, 57]. In fact, a previous study has shown that Bach1 can function as both an activator and a repressor of transcription on the same gene, depending on the cellular context [1]. Therefore, while some evidence suggests that Bach1 inhibition may be an effective therapeutic approach for the treatment of breast cancer [60], the role of Bach1 in cancer growth, progression, and metastasis appears to vary and must be thoroughly characterized for each type of cancer at different stages of tumor progression.

## 6. Bach1 in Hematopoietic Differentiation and Immunity

*β*-Globin gene activation is a crucial step during erythroid differentiation and is impeded by Bach1 [61], which forms a heterodimer with MafK and recruits three transcriptional corepressor complexes nucleosome remodeling and deacetylase (NuRD), switch-insensitive 3a (SIN3A), and switch/sucrose nonfermentable (SWI/SNF) to the locus control region (LCR) of the *β*-globin gene [62]. During erythroid differentiation, Bach1 is replaced by the transcriptional activator p45, which releases the corepressor complexes from the LCR and activates *β*-globin transcription by recruiting coactivators such as CBP, transactivation domain-interacting protein (TIP), and stem cell leukemia (SCL) to the LCR; c-Jun N-terminal kinase (JNK) stabilizes the p45/MafK heterodimer by phosphorylating the Ser157 residue of p45 [62, 63]. Bach1 is also displaced from the *β*-globin LCR (without altering the binding activity of MafK) by intracellular heme [17], and heme biosynthesis requires GATA-1, a master regulator of erythropoiesis that is also transcriptionally suppressed by Bach1. GATA-1 activates globin transcription when heme biosynthesis is normal, but in heme-deficient erythroid cells, Bach1 accumulates and suppresses the GATA-1-mediated transcriptional activation of globin [64]. Heme-induced Bach1 degradation also promotes the transcription factor SPI-C expression in monocytes and the development of bone marrow macrophages [65], and when Bach1 was overexpressed (under the control of the GATA-1 promoter) in transgenic mice, megakaryocyte maturation was significantly impaired and the animals developed thrombocytopenia, likely because Bach1 suppressed the expression of p45-targeted genes such as thromboxane synthase [66]. Bach1 also regulates adipogenesis in primary mouse embryonic fibroblasts by suppressing the expression of peroxisome proliferator-activated receptor (PPAR) *γ* and PPAR*γ*-dependent adipocyte differentiation [67].

Bach1 also has a role in the immune system and autoimmune disease. Bach1 regulates the expression of core macrophage-associated genes, such as aldo-keto reductase family 1 member B10 (Akr1b10), biliverdin reductase B (Blvrb), calcium/calmodulin-dependent protein kinase 1 (Camk1), and glutamate-ammonia ligase (Glul) [68], and both Bach1 and Bach2 promote B-cell development by suppressing myeloid genes in lymphoid progenitor cells [69]. During inflammation, the Bach1/HO-1 pathway regulates osteoclastogenesis, and Bach1 deficiency reduced the severity of osteoarthritis in mice by upregulating HO-1 expression [70, 71]. Bach1 deficiency also impaired the development of antigen-presenting cells (APCs) in mice, which disrupted the T-cell response and partially protected the animals from experimentally induced autoimmune encephalomyelitis [72].

## 7. Conclusions and Future Perspectives

In summary, Bach1 is an important transcription factor that regulates mechanisms involved in ROS production, cell cycle, heme homeostasis, hematopoiesis, and immunity and has a function in cardiovascular disease (e.g., angiogenesis and cardiac hypertrophy) and cancer. Bach1 also regulates adipocyte-related genes, the pentose phosphate pathway, and Wnt/*β*-catenin signaling, which suggests that Bach1 may influence the development and progression of metabolic disease, especially since some Wnt family members and/or downstream targets of Wnt have been linked to insulin sensitivity [73] and diabetes [74]. Other aspects of Bach1 activity that merit continued study include its involvement in epigenetic modifications (e.g., histone methylation and chromatin remodeling). It is clear that Bach1 expression and function differ between different cell types. Future studies should elucidate the role of Bach1 in each type of cancer progression based on clinical studies. Thus, the diverse physiological activity of Bach1 suggests that therapies designed to manipulate Bach1 expression will need to be tightly controlled and tailored for each specific disease state or cell type.

## Abbreviations

Bach1: BTB and CNC homology 1
CNC-bZip: Cap ‘n' Collar and basic region leucine zipper family
HO-1: Oxygenase-1
NQO1: NADPH quinone oxidoreductase 1
MAREs: Maf recognition elements
CP: Cysteine-proline
CLS: Cytoplasmic localization signal
ERK: Extracellular signal-related kinase
Crm1: Chromosome region maintenance 1
IHABP: Intracellular hyaluronic acid-binding protein
Nrf2: Nuclear factor (erythroid-derived 2)-like-2
GCLM: Glutamate cysteine ligase modifier
Keap1: Kelch-like ECH-associated protein 1
Sirt: Sirtuin
TNBS: Trinitrobenzene sulfonic acid
ROS: Reactive oxygen species
GCLC: Glutamate cysteine ligase catalytic subunit
SLC7A11: Solute carrier family 7 member 11
CO: Carbon monoxide
AML: Acute myeloid leukemia
SMCs: Smooth muscle cells
CDK6: Cyclin-dependent kinase 6
CALM1: Calmodulin 1
TFE3: Transcription factor binding to IGHM enhancer 3
EWSR1: EWS RNA-binding protein 1
BCL2L11: BCL2-like 11
HMMR: Hyaluronan-mediated motility receptor
TCF4: Transcription factor 4
LEF1: Lymphoid enhancer-binding factor 1
CBP: CREB-binding protein
HDAC1: Histone deacetylase 1
VEGF: Vascular endothelial growth factor
IL: Interleukin
TAC: Tacrolimus
CXCR4: CXC-chemokine receptor 4
HMGA2: High-mobility group AT-hook 2
MMPs: Matrix metalloproteinases
RKIP: Raf kinase inhibitory protein
MAFG: Musculoaponeurotic fibrosarcoma oncogene homolog G
CHD8: Chromodomain helicase DNA-binding protein 8
TMZ: Temozolomide
MGMT: Methylguanine DNA methyltransferase
TKT: Transketolase
NuRD: Nucleosome remodeling and deacetylase
SIN3A: Switch-insensitive 3a
SWI/SNF: Switch/sucrose nonfermentable
LCR: Locus control region
JNK: c-Jun N-terminal kinase
PPAR: Peroxisome proliferator-activated receptor
Akr1b10: Aldo-keto reductase family 1 member B10
Blvrb: Biliverdin reductase B
Camk1: Calcium/calmodulin-dependent protein kinase 1
Glul: Glutamate-ammonia ligase
APCs: Antigen-presenting cells.
